# Optimized strategy of switching to tenofovir alafenamide fumarate treatment for nucleos(t)ide analogue experienced patients with chronic hepatitis B

**DOI:** 10.3389/fmed.2025.1592998

**Published:** 2025-11-14

**Authors:** Xuan Hu, Yin Kong, Yuanyuan Liu, Tianfu Liu, Aidi Ma, Yongfang Li, Yaping Zhang, Juan Li, Lingyi Zhang, Guangming Li

**Affiliations:** 1 Department of Gastroenterology, Anhui No.2 Provincial People’s Hospital, Hefei, Anhui, China; 2 Department of Hepatology, Lanzhou University Second Hospital, Lanzhou, Gansu, China

**Keywords:** chronic hepatitis B, tenofovir alafenamide fumarate, antiviral treatment, low-level viremia, partial virological response

## Abstract

**Background:**

This study aimed to evaluate the efficacy and safety of tenofovir alafenamide fumarate (TAF) in nucleos(t)ide analogue (NA)-experienced patients with chronic hepatitis B (CHB) who exhibited partial virological response (PRT) or low-level viremia (LLV).

**Methods:**

This single-center, retrospective, real-world study enrolled NA-experienced CHB patients who were switched to TAF treatment. Patients were categorized into the PRT (HBV DNA > 2,000 IU/mL) or LLV (20 IU/ mL < HBV DNA ≤ 2,000 IU/mL) groups according to baseline HBV DNA levels. The dynamic changes in HBV DNA, HBsAg, HBeAg, ALT, and APRI were analyzed after switching to TAF.

**Results:**

A total of 91 CHB patients with prior NA treatment and detectable HBV DNA after at least 48 weeks of therapy were enrolled and subsequently switched to TAF. Among them, 24 patients had PRT, and 67 patients had LLV. The complete virological response rate (HBV DNA < 20 IU/mL) in the PRT group was 29.1% at week 24 and 75.0% at week 48; in the LLV group, it was 76.1% and 88.1%, respectively. Both groups showed a decline in HBeAg levels from baseline to week 24 and 48. In the PRT group, HBsAg levels decreased by 9.0% and 5.0% at week 24 and 48, respectively; in the LLV group, the reductions were 2.1% and 3.6%. The ALT normalization rate increased by 24.2% at week 48 compared with baseline. Additionally, eGFR levels improved after switching to TAF. No serious adverse events (SAEs) or deaths related to adverse events were observed.

**Conclusion:**

This real-world study suggests that switching to TAF is an effective and well-tolerated therapeutic strategy for NA-experienced CHB patients with PRT or LLV, offering a promising approach for treatment optimization.

## 1 Introduction

It is estimated that approximately 86 million people in China are living with persistent hepatitis B virus (HBV) infection, among whom around 28 million are diagnosed with chronic hepatitis B (CHB) (1, 2). Persistent HBV infection significantly increases the risk of developing liver cirrhosis (LC) and hepatocellular carcinoma (HCC) (3). A high serum HBV-DNA load is an important factor in the development of HCC in patients with persistent HBV infection (4, 5). Meanwhile, serum hepatitis B surface antigen (HBsAg) levels are also an important determinant of the risk of HCC in HBV-infected patients, especially in CHB patients with serum HBsAg levels of 1,000 IU/mL or higher, where the cumulative incidence of HCC is significantly higher than in patients with serum HBsAg levels < 1,000 IU/mL (6). Therefore, reducing serum HBV DNA and HBsAg levels is key to preventing the progression of chronic serious complications such as LC and HCC in patients with persistent HBV infection.

Currently, nucleoside analogue/nucleotide analogue (NA) treatment can significantly reduce HBV DNA levels. However, low-level viremia (LLV) remains a common issue in some patients undergoing long-term NA therapy. For instance, some patients treated with entecavir (ETV) are still in a state of LLV and poor response (PRT) to antiviral therapy (7). Moreover, ETV has limited efficacy in reducing serum HBsAg levels (8, 9). Studies have shown that the incidence of disease progression and adverse outcomes in this population is higher than in patients with complete virologic response (10); and long-term use of tenofovir disoproxil fumarate (TDF) can cause bone and kidney damage (11, 12).

Tenofovir alafenamide fumarate (TAF), approved in China in November 2018, is recommended as a first-line antiviral therapy in the 2019 edition of the Chinese guidelines for the prevention and treatment of chronic hepatitis B (13). The guidelines also recommend TAF as a switching strategy for CHB patients with LLV or PRT. Therefore, this single-center, retrospective real-world study aims to evaluate the efficacy and safety of switching to TAF in treatment-experienced CHB patients with LLV or PRT in western China.

## 2 Materials and methods

### 2.1 Patients

This retrospective study included 95 treatment-experienced patients from the Department of Liver Diseases at the Second Hospital of Lanzhou University, between January 2019 and January 2021. All patients met the following inclusion criteria: (1) chronic HBV infection was diagnosed in accordance with the criteria of “Chinese guidelines for the prevention and treatment of chronic hepatitis B” (14); (2) prior NA treatment duration ≥ 48 weeks; (3) HBV DNA > 20 IU/mL; (4) age ≥ 18 years.

Exclusion criteria included the following: (1) co-infection with other hepatitis viruses (hepatitis A, C, D, or E); (2) presence of other liver diseases, including alcoholic liver disease, non-alcoholic fatty liver disease, drug-induced liver injury, autoimmune liver disease, or inherited metabolic disorders; (3) severe liver conditions, such as acute or chronic liver failure; (4) coexisting severe cardiovascular disease, chronic renal failure [estimated glomerular filtration rate (eGFR) < 45 mL/min/1.73 m^2^], or hematologic disorders; (5) co-infection with human immunodeficiency virus (HIV); (6) pregnancy.

### 2.2 Measurements

Serum HBV DNA were measured by Roche Cobas AmpliPrep/CobasTaqMan (lower limit of detection is 20 IU/mL), serum HBsAg and HBeAg quantification were measured by Roche Cobas AmpliPrep/CobasTaqMan, serum liver biochemistry indicators were detected by Roche Cobas 8000 automatic biochemical instrument (normal range of ALT: 0–50 U/L, normal range of AST: 0–40 U/L), blood test were detected by Mindray BC-6900 blood cell analyzer, eGFR was determined by the Cockcroft-Gault method.

### 2.3 Study subgroups

#### 2.3.1 PRT group

CHB patients showed serum HBV DNA > 2,000 IU/mL with NA treatment for at least 48 weeks; LLV group: CHB patients showed serum HBV DNA < 2,000 IU/mL but still detectable (detection limit of 20 IU/mL) with NA treatment for at least 48 weeks.

### 2.4 Clinical outcomes

#### 2.4.1 Primary endpoints

(1) Complete response (CVR) defined as serum HBV DNA < 20 IU/mL at 24 ± 2w and 48 ± 2w of TAF treatment; (2) subgroup analysis of the rate of complete virological response (CVR); the degree of serum HBV DNA level decreased from baseline in PRT and LLV groups; (3) the degree of serum HBeAg decreased from baseline and the rate of HBeAg negative/seroconversion in all patients; (4) subgroup analysis of the degree of serum HBeAg decreased from baseline and the rate of HBeAg negative/seroconversion in the PRT and LLV groups.

#### 2.4.2 Secondary endpoints

(1) The degree of serum HBsAg decreased from baseline and the rate of HBsAg negative/seroconversion in all patients; (2) subgroup analysis of degree of serum HBsAg decreased from baseline and the rate of HBsAg negative/seroconversion in the PRT and LLV groups; (3) comparing the decreased degree of ALT and ALT normalization rate; (4) comparison of the aspartate aminotransferase to platelet ratio index [APRI = AST/ULN × 100/PLT (×10^9^/L)].

Primary safety endpoint: the occurrence of adverse reactions (AEs) during treatment during the treatment.

Secondary safety endpoint: the changes in renal function, measured by eGFR.

### 2.5 Statistical analysis

SPSS 25.0 software was applied for the statistical analysis of the data. The *χ*^2^ test and Fisher’s exact test were used for the counting data. Use the median and upper and lower quartiles [M (Q1, Q3)] for data that does not conform to the normal distribution. The differences between the three groups were compared using the Friedman rank sum test, and the differences between the two groups were compared using the Wilcoxon rank sum test. *p* < 0.05 indicates that the difference is statistically significant, We adjusted the *p*-values for multiple comparisons using the Bonferroni method, *p* < 0.05 indicates that the difference is statistically significant. Use GraphPad Prime 8.0 to draw graphics.

## 3 Results

### 3.1 Baseline characteristics

A total of 95 CHB treatment-experienced patients who met the criteria were enrolled, of whom 4 dropped out of the study. The number of patients in the PRT and LLV groups was 24 and 67, and the baseline characteristics of patients were showed in Table 1. The LLV group had a lower proportion of male sex, lower HBV DNA levels, lower HBeAg levels, lower ALT levels, and a lower APRI score, while a higher proportion of ETV experienced treatment (*p* < 0.05).

**Table 1:** Baseline characteristics of enrolled patients.

Baseline characteristics	Total (*n* = 91)	PRT group (*n* = 24)	LLV group (*n* = 67)	*p*-value
Age, years	38.0 ± 10.0	38.1 ± 11.5	39.5 ± 9.6	0.610
Male sex, *n* (%)	56 (61.5)	19 (79.2)	37 (52.2)	0.039
Comorbidity				
HCC, *n* (%)	1 (1.1)	1 (4.2)	0 (0)	NA
Type 2 diabetes, *n* (%)	3 (3.3)	2 (8.3)	1 (1.5)	NA
Hypertension, *n* (%)	2 (2.2)	1 (4.2)	1 (1.5)	NA
CKD, *n* (%)	1 (1.1)	1 (4.2)	0 (0)	NA
Hypothyroidism, *n* (%)	1 (1.1)	1 (4.2)	0 (0)	NA
Experienced ETV therapy, *n* (%)	65 (71.4)	8 (33.3)	57 (85.1)	<0.001
HBV DNA, log IU/mL	2.2 (1.7, 3.6)	6.1 (4.2, 7.3)	1.9 (1.8, 2.4)	<0.001
HBsAg, log IU/mL	3.6 (3.3, 3.9)	3.6 (3.2, 4.2)	3.6 (3.3, 3.9)	0.449
HBeAg, log IU/mL	0.7 (−0.9, 2.0)	1.8 (0.4, 2.9)	0.5 (−1.0, 1.8)	0.031
HBeAg positive, *n* (%)	62 (68.1)	19 (79.2)	43 (64.2)	0.176
ALT, U/L	35.0 (20.0, 73.0)	75. 0 (46.0, 146.3)	25.0 (18.0, 49.0)	<0.001
eGFR, mL/min/1.73m^2^	105.6 (98.6, 112.6)	104.4 (94.6, 110.8)	106.6 (100.0, 113.4)	0.397
APRI score	0.5 (0.3, 1.0)	1.0 (0.5, 2.1)	0.4 (0.3, 0.7)	0.002

### 3.2 Virologic response

**Figure 1:**
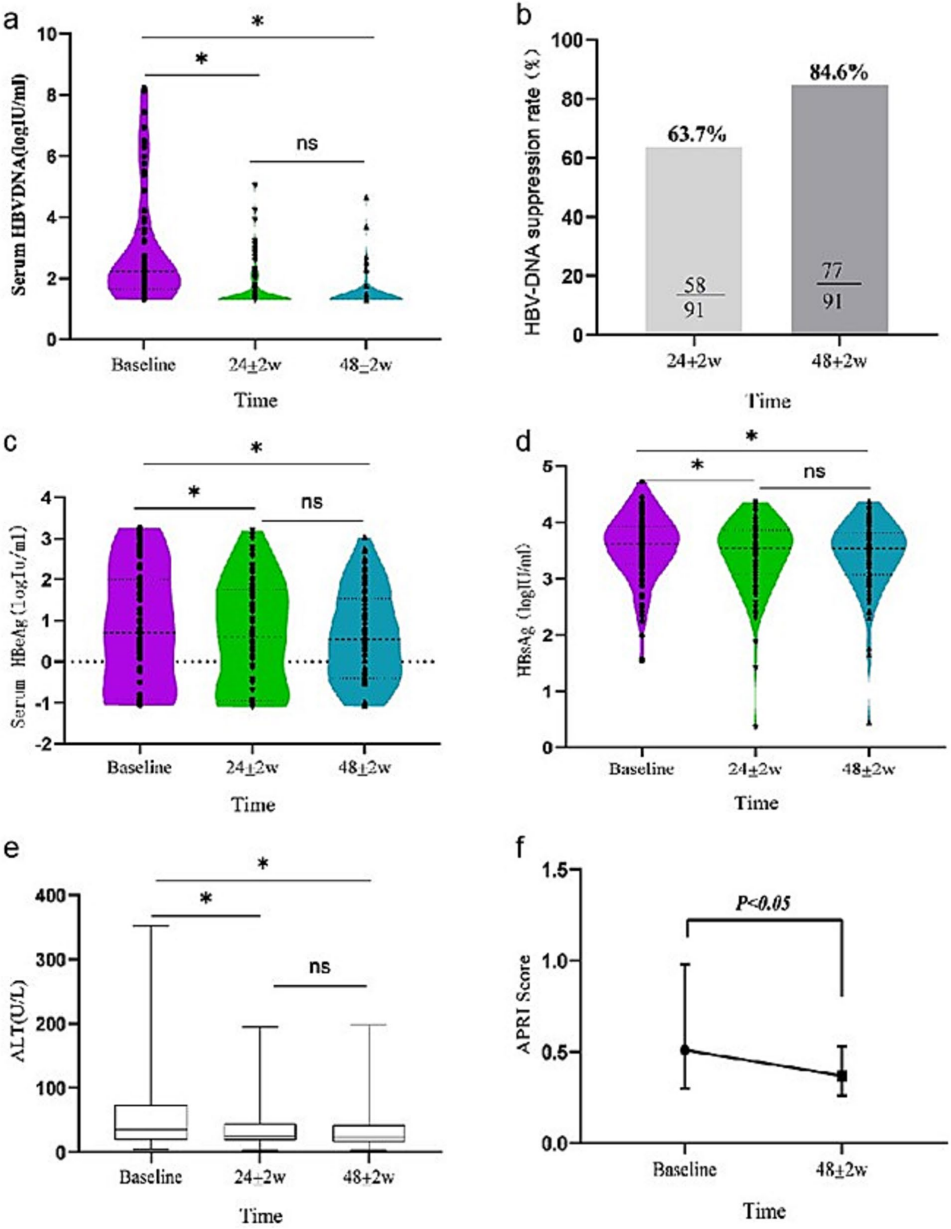
Dynamic change of virological [**(a)** serum HBV DNA level, **(b)** HBV DNA suppression rate], serological [**(c)** serum HBeAg level, **(d)** serum HBsAg level], and biochemical biomarker [**(e)** ALT level, **(f)** APRI index] response during 24 ± 2w and 48 ± 2w of TAF therapy in all patients.

Compared with baseline, the levels of HBV DNA at 24 ± 2w and 48 ± 2w showed a significant downward trend (*χ*^2^ = 116.462, *p* < 0.001) after switching to TAF treatment, among which, the differences were statistically significant at 24 ± 2w and 48 ± 2w compared with baseline (*Z* = −7.145, *p* < 0.001 adjusted *p*-value < 0.001; *Z* = −7.948, *p* < 0.001, adjusted *p*-value < 0.001). The differences were statistically significant 48 ± 2w at compared with 24 ± 2w (adjusted *p*-value < 0.05). The decrease degree of serum HBV DNA at 24 ± 2w and 48 ± 2w compared with baseline was 44.0 and 51.8% (Table 2 and Figure 1a). The CVR rates were 63.7 and 84.6% at 24 ± 2w and 48 ± 2w (Table 2 and Figure 1b).

**Table 2:** Virological and serological response of TAF treatment.

Treatment response	Total (*n* = 91)	PRT group (*n* = 24)	LLV group (*n* = 67)	*p*-value
HBV DNA (log_10_ IU/mL)				
Baseline	2.2 (1.7, 3.6)	6.1 (4.2, 7.3)	2.2 (1.8, 2.4)	<0.001
24 ± 2w	1.3 (1.3, 1.7)^**^	1.8 (1.3, 2.8)^**^	1.3 (1.3, 1.9)^**^	0.017
48 ± 2w	1.3 (1.3, 1.3)^**^	1.3 (1.3, 1.3)^**^	1.3 (1.3, 1.3)^**^	<0.001
Decrease degree of HBV DNA (%)				
Baseline	—	—	—	
24 ± 2w	44.0	64.8	22.6	
48 ± 2w	51.8	72.0	30.0	
CVR rate (*n*, %)				
Baseline	—	—	—	
24 ± 2w	58 (63.7)	7 (29.2)^*^	51 (76.1)^*^	<0.001
48 ± 2w	77 (84.6)	18 (75.0)^*^	59 (88.1)^*^	0.185
HBeAg (log_10_ IU/mL)				
Baseline	0.7 (−0.9, 2.0)	1.8 (0.4, 2.9)	0.5 (−1.0, 1.8)	0.031
24 ± 2w	0.6 (−0.9, 1.8)^*^	1.6 (0.1, 2.0)^*^	0.4 (−1.0, 1.5)	0.021
48 ± 2w	0.6 (−0.4, 1.5)^*^	1.1 (0.3, 1.8)^*^	0.4 (−0.4, 1.3)	0.058
Decrease degree of HBeAg (%)				
Baseline	—	—	—	
24 ± 2w	22.2	17.7	44.6	
48 ± 2w	26.7	41.0	47.3	
HBsAg (log_10_ IU/mL)				
Baseline	3.6 (3.3, 3.9)	3.6 (3.2, 4.2)	3.6 (3.3, 3.9)	0.449
24 ± 2w	3.5 (3.1, 3.9)^**^	3.4 (2.9, 3.7)^*^	3.6 (3.1, 3.9)^**^	0.211
48 ± 2w	3.5 (3.1, 3.8)^**^	3.5 (3.0, 3.8)^*^	3.6 (3.1, 3.9)^**^	0.836
Decrease degree of HBsAg (%)				
Baseline	—	—	—	
24 ± 2w	4.0	9.0	2.1	
48 ± 2w	4.1	5.0	2.1	

^*^Denotes *p* < 0.05 compared with baseline level. ^**^Denotes *p* < 0.001 compared with baseline level.

**Figure 2:**
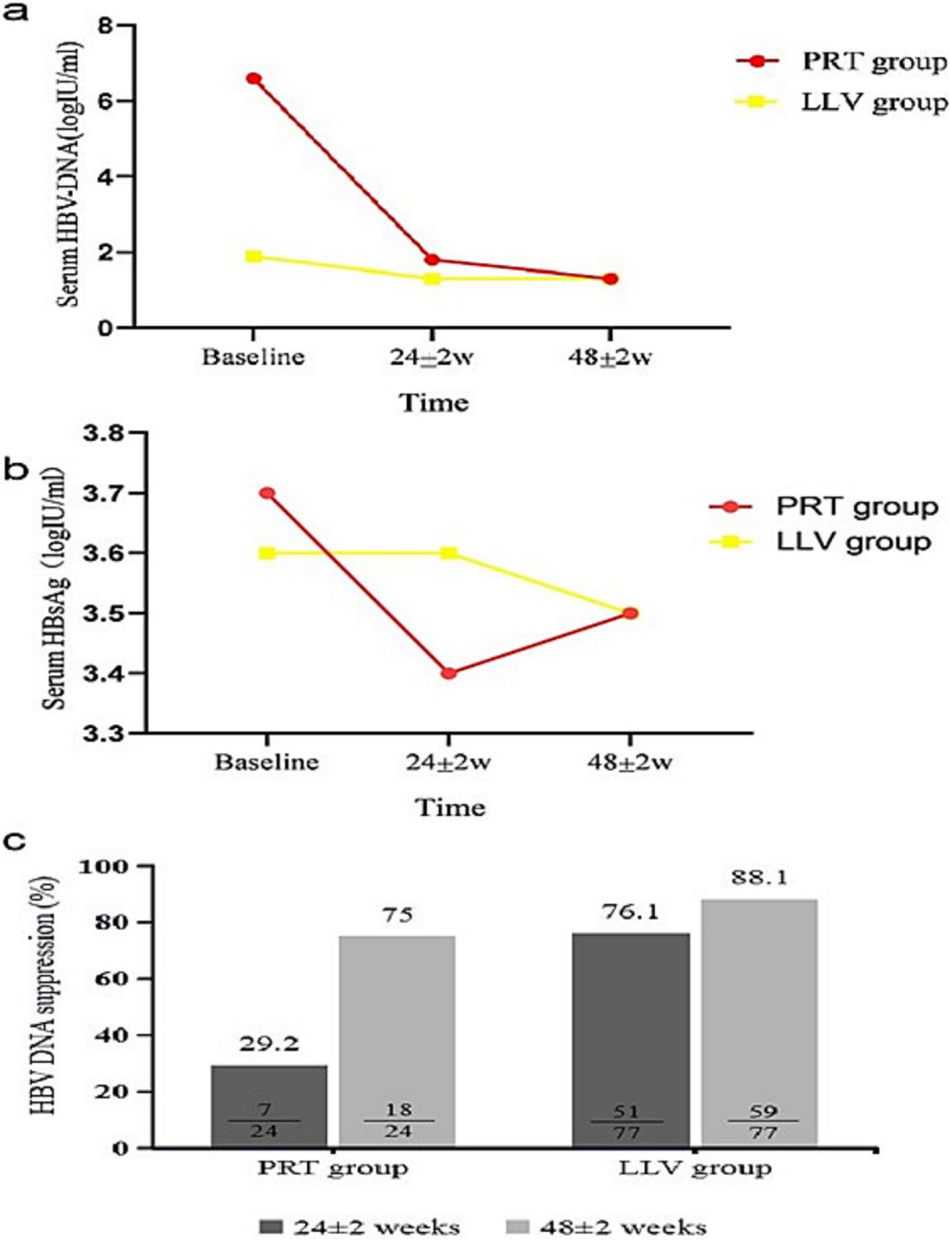
Dynamic change of virological [**(a)** serum HBV DNA level, **(c)** HBV DNA suppression rate] and serological biomarker [**(b)** serum HBsAg level] response during 24 ± 2w and 48 ± 2w of TAF therapy in PRT group and LLV group.

Subgroup analysis showed that, in the PRT group, compared with baseline, HBV DNA was reduced significantly in both timepoints at 24 ± 2w and 48 ± 2w (*χ*^2^ = 43.888, *p* < 0.001) (Table 2 and Figure 2a). The CVR rates were 29.1 and 75.0% at 24 ± 2w and 48 ± 2w of switch therapy (Table 2 and Figure 2c). In the LLV group, HBV DNA level was significantly decreased at 24 ± 2w and 48 ± 2w (*χ*^2^ = 87.484, *p* < 0.001) (Table 2 and Figure 2a). The CVR rates were 76.1 and 88.1% at 24 ± 2w and 48 ± 2w (Table 2 and Figure 2c). As of the HBV DNA decreased degree comparison, in the PRT group, HBV DNA decreased degrees were 64.8 and 72.0% at 24 ± 2w and 48 ± 2w. In the LLV group, the decreased degree was 22.6 and 30.0% (Table 2).

### 3.3 Serological response

In all enrolled patients, HBeAg showed a decreasing trend at 24 ± 2w and 48 ± 2w (*χ*^2^ = 8.715, *p* = 0.013) compared with baseline. The differences were statistically significant at 24 ± 2w and 48 ± 2w compared with baseline (*Z* = −3.688 *p* < 0.001, adjusted *p*-value = 0.049; *Z* = −2.581 *p* = 0.010 adjusted *p*-value = 0.079). The differences were not statistically significant 48 ± 2w at compared with 24 ± 2w (adjusted *p*-value = 0.042). The decreased degrees of HBeAg were 22.2 and 26.7% at 24 ± 2w and 48 ± 2w. HBeAg negative rates were 3.2 and 8.1% at 24 ± 2w and 48 ± 2w (Table 2 and Figure 1c).

Subgroup analysis showed that (Table 2), in the PRT group, HBeAg was decreased significantly at 24 ± 2w and 48 ± 2w (*χ*^2^ = 13.000, *p* = 0.002) compared with baseline. At 24 ± 2w and 48 ± 2w, HBeAg decreased by 17.7 and 41.0%. In the LLV group, HBeAg were also decreased significantly (*χ*^2^ = 3.630, *p* = 0.163), while HBeAg decreased degree were 44.6 and 47.3% at 24 ± 2w and 48 ± 2w.

As for the HBsAg response, the change in serum HBsAg after switching to TAF treatment was significant (*χ*^2^ = 28.424, *p* < 0.001). Compared with baseline, serum HBsAg decreased by 4.0 and 4.1% in 24 ± 2w and 48 ± 2w. Only one patient experienced HBsAg clearance, HBsAg rate was 1.1% (Table 2 and Figure 1d).

Subgroup analysis showed that (Table 2 and Figure 2b), the differences of HBsAg were statistically significant in the PRT group (*χ*^2^ = 13.083, *p* < 0.001). Compared with baseline, HBsAg decreased by 9.0 and 5.0% at 24 ± 2w and 48 ± 2w. In the LLV group, the differences in HBsAg at 24 ± 2w and 48 ± 2w were both statistically significant (*Z* = −4.446 *p* < 0.001, *Z* = −6.513, *p* < 0.001) compared with baseline. HBsAg decreased degrees were 2.1 and 3.6% at 24 ± 2w and 48 ± 2w.

### 3.4 Biochemical change

All included patients showed significant changes in ALT levels after switching to TAF treatment (Table 2 and Figure 1e). Compared with baseline, ALT levels decreased at 24 ± 2w and 48 ± 2w (*χ*^2^ = 28.424, *p* < 0.001). The differences were statistically significant at 24 ± 2w and 48 ± 2w compared with baseline (adjusted *p*-value < 0.005; adjusted *p*-value < 0.05). The differences were not statistically significant 48 ± 2w at compared with 24 ± 2w (adjusted *p*-value > 0.05). After 48 ± 2w of TAF treatment, the ALT normalization rate increased by 24.2% from baseline.

### 3.5 APRI change

The APRI scores at 24 ± 2w and 48 ± 2w were 0.5 (0.3, 1.0) and 0.4 (0.3, 0.5). There was a statistically significant decrease in APRI scores compared to baseline at 48 ± 2w of TAF replacement treatment (*Z* = 3.495, *p* = 0.001) (Figure 1f).

### 3.6 Safety

The median eGFR at baseline, 24 ± 2w and 48 ± 2w were 105.6 (98.6, 112.6), 104.8 (98.3, 112.5), and 108.8 (101.6, 119.3) mL/min/1.73 m^2^. There were significantly differences in eGFR at the three observation time points (*χ*^2^ = 20.738, *p* < 0.001), while eGFR levels were significantly higher at 48 ± 2w compared to 24 ± 2w (*Z* = −4.470, *p* < 0.001 adjusted *p*-value < 0.0001).

The overall incidence of adverse reactions at 48 weeks of TAF therapy was 3.2% (3/91), the main adverse reactions including: fatigue (1.1%), loss of appetite (1.1%), and facial erythema (1.1%). There was one patient who discontinued the drug at 12 weeks due to facial erythema and subsided after switching to TDF. No SAEs were observed in this study.

## 4 Discussion

In the guidelines of all countries, HBV DNA replication should be greatly inhibited to prevent disease progression and HCC occurrence, which lead to improving quality of life and prolonging the survival time of CHB patients, which are the main goals of antiviral therapy (13, 14). However, in real-world clinical practice, only a small portion of patients had detectable HBV DNA after long-term antiviral treatment. According to the Chinese guidelines for the prevention and treatment of chronic hepatitis B (2019 version), patients with HBV DNA levels >2,000 IU/mL after 48 weeks of first-line nucleos(t)ide analogue therapy—after excluding poor adherence and testing error—are considered to have a “poor response” and should be considered for treatment adjustment (13). The American Association for the Study of Liver Diseases (AASLD) 2018 update defines low-level viremia (LLV) as detectable HBV DNA < 2000 IU/mL (detection limit of 10 IU/mL) after 48 weeks of antiviral therapy, but it does not recommend immediate treatment modification in these patients (14). More recently, the European Association for the Study of the Liver (EASL) 2025 guidelines emphasise that patients with persistent detectable HBV DNA after prolonged therapy should undergo a comprehensive assessment—adherence, resistance testing, fibrosis progression—and consideration of treatment switch or intensification, especially in the presence of liver damage or other risk factors (15). These evolving definitions and recommendations across guidelines underline the need for further clinical evidence to guide management of PRT and LLV.

Recently, there were many studies focused on the NA switch to the TAF strategy. Ogawa et al. (16) found that CHB patients treated with ETV switched to TAF for 48 weeks, the virological response rate increased from 75.9 to 96.9%, and eGFR also improved (+0.40 mL/min/1.73m^2^). In the study of Nguyen et al. (17) found that after an average of 6 years on ETV, switching to TAF treatment increased CVR from 91.9 to 97.2% after 96 weeks. These studies suggested that TAF has promising antiviral efficacy in NA experienced CHB patients. The results of our study can further illustrate that the switch to TAF treatment in CHB treated patients could further inhibit the replication of HBV DNA and increase the CVR rate to 63.7% in 24 ± 2w and 84.6% at 48 ± 2w.

In the study of Li et al. (18), CHB patients with persistent or intermittent LLV status after ETV treatment who switched to TAF could increase the CVR rate by 62.7% for 24 weeks, compared with continuing ETV group, the CVR rate was only 9.3%, suggesting that LLV patients have a higher possibility to achieve CVR after switching to TAF treatment. In our study, we found that the CVR rate was 76.1% in the LLV group after 24 ± 2 weeks of switching to TAF therapy, which was higher than the study by Li et al. (18), and the CVR rate of 48 ± 2 weeks in the LLV group could be further increased to 88.1%. In the PRT group, the CVR significantly increased at 48 ± 2 weeks compared with 24 ± 2 weeks (26.2, 75.0%). Therefore, CHB patients with PRT or LLV status could both benefit from switching to TAF therapy.

The serological response was another major efficacy indicator of antiviral treatment. After switching to TAF therapy, the decreased degree of serum HBeAg was 22.2% for 24 ± 2 weeks, and gradually increased to 26.7% at 48 ± 2 weeks. In the PRT and LLV groups, we found that the decrease degree in the LLV group at 24 ± 2 weeks (44.6%) was significantly higher than that in the PRT group (17.7%), while the decreased degree in the PRT group increased rapidly to 41.0% at 48 ± 2 weeks. Lampertico et al. (19) reported that of 78 HBeAg positive patients who were treatment experienced, after switching to TAF, 6 patients (8%) had HBeAg clearance at 48 weeks. Our study observed a similar result, the HBeAg clearance rate was 8.1% after 48 ± 2 weeks of TAF treatment.

In the study by Uchida et al. (20), the decrease in HBsAg during treatment with ETV in CHB patients was approximately 0.041 log_10_ IU/mL, and the decreased degree was increased to 0.068 log_10_ IU/mL after switching to TAF for 48 weeks. Meanwhile, the effect of TAF in reducing serum HBsAg was more significant in patients with baseline HBsAg < 800 IU/mL. In our study, the decreased degree of serum HBsAg was 4.1% after switching to TAF at 48 ± 2 weeks. One patient has achieved HBsAg loss. Subgroup analysis suggested that some patients in the PRT group showed an increase in HBsAg during TAF treatment, but the magnitude of the increase was small, which was considered a fluctuation caused by the test reagent of HBsAg. It can also be affected by the immune status of the host (21). Many factors can influence the level of HBsAg and HBeAg, such as drugs, immune status, which can change during long-term suppression of HBV. Further studies are needed to confirm the changes in serum HBsAg and HBeAg in patients who consistently obtained CVR after switching to TAF treatment.

The serum ALT level reflected the degree of hepatocellular damage. In this study, the ALT normalization rate was significantly increased after 48 ± 2 weeks of TAF treatment, which was similar to the results of the TAF global phase III clinical trial (22). Our further analysis revealed that ALT levels were significantly lower than baseline at week 24 ± 2w, suggesting that TAF can significantly alleviate hepatocyte inflammation while rapidly suppressing viral replication. In addition, we also observed that the APRI score was significantly lower after 48 ± 2 weeks of TAF treatment.

TAF has a better renal safety profile compared to TDF (22, 23). Kaneko et al. (24) found that in CHB patients who developed renal impairment during TDF treatment, the renal function could be improved after switching to TAF, the eGFR levels increased significantly at week 4 and week 24 of TAF treatment. Lampertico et al. (19) also found that TAF improved renal tubular function in CHB patients. In our study, most patients had normal renal function at baseline. Although eGFR levels showed a mild numerical increase after switching to TAF for 48 ± 2 weeks, no renal adverse events were observed. Given the normal baseline renal function, this slight change in eGFR is likely of limited clinical significance, but it further supports the favorable renal safety profile of TAF in treatment-experienced CHB patients.

In addition, as this was a retrospective, single-arm analysis, no concurrent control group was included. The primary objective was to compare virological efficacy among patients with different baseline viral statuses. However, we have incorporated historical control data from our previous LLV cohort, in which the CVR rate at 48 ± 2 weeks without switching was only 16.2% (25), to provide context for our findings.

There are some limitations to our study. Firstly, the patients enrolled in this study were from a single center, resulting in a small number of patients in each subgroup, which may affect the effectiveness of the observed clinical results. Secondly, the observation period of this study was 48 ± 2 weeks, which was insufficient to fully evaluate the efficacy and safety of long-term antiviral treatment with TAF, and further follow-up was needed. Finally, large-scale, multicenter, randomized controlled trials were needed to explore the efficacy and safety of TAF in real-world settings to provide more valuable evidence for the optimized strategy of antiviral regimens for CHB experienced with PRT and LLV.

## 5 Conclusion

In conclusion, our study observed the efficacy and safety of the TAF therapy in NA-experienced CHB patients with PRT or LLV status in western China and found that switching to TAF could further suppress HBV DNA replication, increase the CVR rate, reduce the HBsAg and HBeAg levels, improve ALT normalization, and renal function. Switching to TAF treatment is a promising choice for an optimized strategy of NA experienced CHB patients with PRT or LLV.

## Data Availability

The raw data supporting the conclusions of this article will be made available by the authors, without undue reservation.

## Author’s note

Part of the result were presented in the form of poster at the 2022 Annual Meeting of the Asian Pacific Association for the Study of the Liver (APASL).
